# Traditional Chinese medicine for hypertrophic scars—A review of the therapeutic methods and potential effects

**DOI:** 10.3389/fphar.2022.1025602

**Published:** 2022-10-10

**Authors:** Daqin Chen, Qiannan Li, Huimin Zhang, Fang Kou, Qiang Li, Chunming Lyu, Hai Wei

**Affiliations:** ^1^ Institute of Interdisciplinary Integrative Medicine Research, Shanghai University of Traditional Chinese Medicine, Shanghai, China; ^2^ Shuguang Hospital Affiliated to Shanghai University of Traditional Chinese Medicine, Shanghai, China; ^3^ Experiment Center for Science and Technology, Shanghai University of Traditional Chinese Medicine, Shanghai, China; ^4^ Qinghai Province Key Laboratory of Tibetan Medicine Pharmacology and Safety Evaluation, Northwest Institute of Plateau Biology, Chinese Academy of Sciences, Shanghai, China

**Keywords:** hypertrophic scar (HS), traditional Chinese medicine (TCM), anti-HS mechanism, new drug delivery system, therapeutic strategy

## Abstract

Hypertrophic scar (HS) is a typical pathological response during skin injury, which can lead to pain, itching, and contracture in patients and even affect their physical and mental health. The complexity of the wound healing process leads to the formation of HS affected by many factors. Several treatments are available for HS, whereas some have more adverse reactions and can even cause new injuries with exacerbated scarring. Traditional Chinese Medicine (TCM) has a rich source, and most botanical drugs have few side effects, providing new ideas and methods for treating HS. This paper reviews the formation process of HS, the therapeutic strategy for HS, the research progress of TCM with its relevant mechanisms in the treatment of HS, and the related new drug delivery system of TCM, aiming to provide ideas for further research of botanical compounds in the treatment of HS, to promote the discovery of more efficient botanical candidates for the clinical treatment of HS, to accelerate the development of the new drug delivery system and the final clinical application, and at the same time, to promote the research on the anti-HS mechanism of multiherbal preparations (Fufang), to continuously improve the quality control and safety and effectiveness of anti-HS botanical drugs in clinical application.

## 1 Introduction

The scar is the abnormal product of wound healing. Its formation and development are closely related to the tissue repair process and involve many regulatory pathways. The formation of a hypertrophic scar (HS) affects the appearance and function of patients’ skin and brings severe physiological and psychological problems to patients (Li W. et al., 2021; Li et al., 2021c). More than 100 million new cases of scarring occur every year in developing countries (Zhang et al., 2018). The overall incidence of HS after skin trauma is 40%–70%. The incidence of HS after burn is as high as 80% (Wei et al., 2021), and the market for anti-scar drugs is more than $12 billion annually (Sen et al., 2009). The treatment of HS is a challenging topic in the field of burn, plastic surgery, and rehabilitation (Mousavizadeh et al., 2021). Wound healing can be divided into four stages: hemostasis, inflammation, proliferation, and remodeling. The mechanisms involved in scar formation in each stage are also different (Bowers and Franco, 2020). At present, there are surgical treatment, physical therapy (Zhang et al., 2019), laser therapy (Tan et al., 2021), cryotherapy (Tunca et al., 2019), and drug therapy (Bi et al., 2019) for HS. Each treatment has its advantages and disadvantages (Elsaie, 2021). However, effective prevention and treatment measures were still limited. As a kind of drug therapy, the botanical compounds extracted from TCM, with a rich source, showed excellent potential for scar treatment. So far, the TCM commercial drugs for the treatment of HS, including the asiaticoside tablets and asiaticoside cream ointment (Shanghai Shyndec Pharmaceutical Co., Ltd., license No. Z31020564 and Z20054146), external scar antipruritic softening ointment (Chengdu Dongyang Baixin Pharmaceutical Co., Ltd., license No. Z20050438 ), and moist burn ointment (Shantou Meibao Pharmaceutical Co., Ltd., license No. Z20000004 ), are commonly used in the clinic. The formation process of HS, the therapeutic methods for HS, the effects of TCM on HS, the HS inhibitory mechanism of TCM, and the new drug delivery system of TCM for HS were reviewed in the paper, aiming to give insight into the research on the anti-HS mechanism of botanical drugs, to discover new anti-HS drug from TCM, to promote the development of new drug delivery system of anti-HS botanical drugs, to assist in developing effective experimental and clinical strategies for the treatment of HS using TCM, and to eventually optimize the clinical application of anti-HS botanical drugs based on the safety, efficacy, and rationality. So far, there are few comprehensive reviews on TCM for treating HS.

## 2 The formation process of HS

**FIGURE 1:**
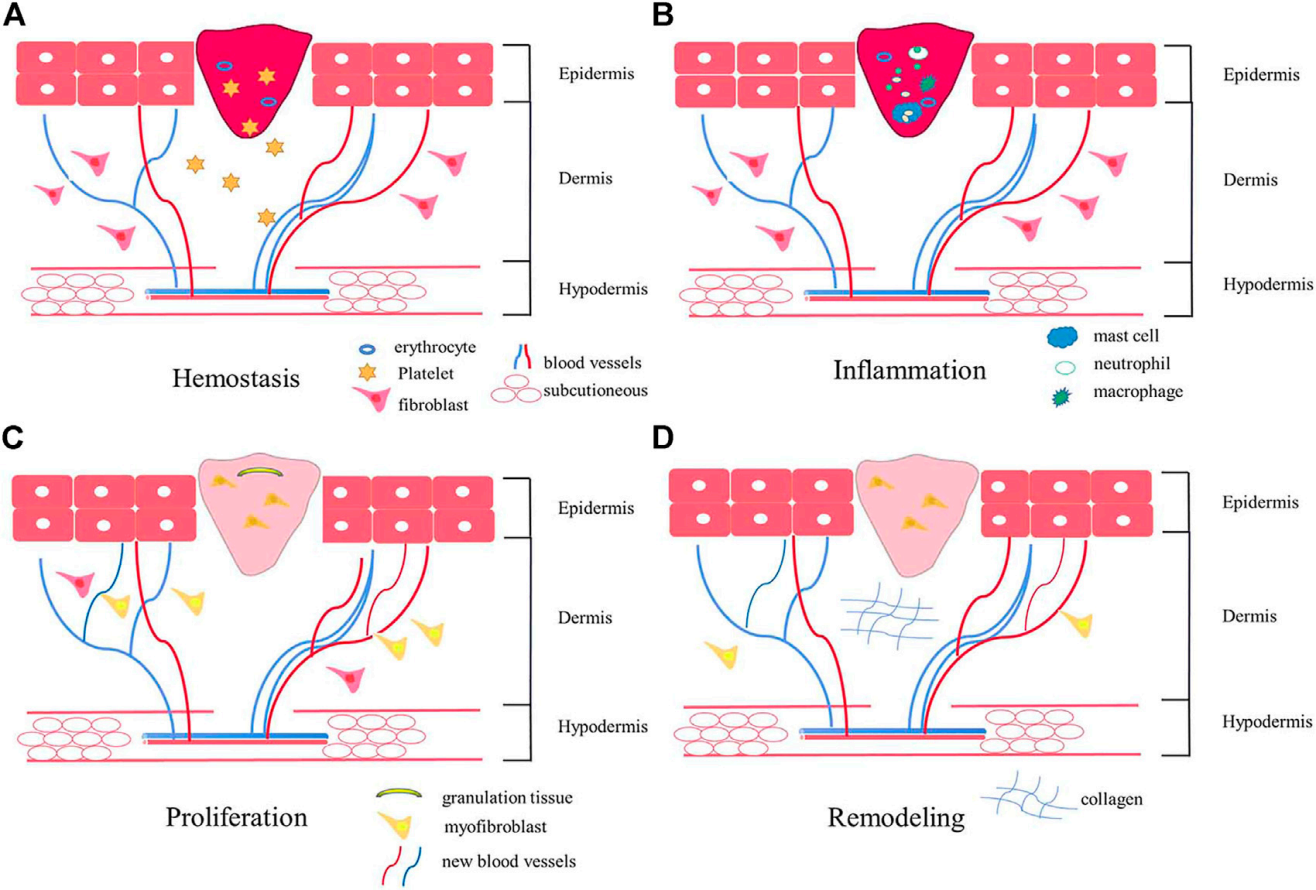
The schematic diagram of the wound healing process. Wound healing includes four stages: hemostasis **(A)**, inflammation **(B)**, proliferation **(C)**, and remodeling **(D)**. After the injury, platelets aggregate and release soluble media to initiate hemostasis. Shortly thereafter, inflammation begins, and macrophages accumulate and migrate to the wound, promoting phagocytosis of bacteria and injuring tissues. Angiogenesis occurs at the proliferation stage. In the final remodeling stage, wound tensile strength increases.

Each wound must pass through the wound-healing process to heal appropriately. The wound-healing process comprises four overlapping stages: hemostasis, inflammation, proliferation, and remodeling (as shown in Figure 1) (Veith et al., 2019; Orlowski et al., 2020). ①. Immediately after injury, hemostasis begins, where clotting pathways are triggered, a temporary fibrin matrix is formed, and associated cells migrate to the injury site (Subramaniam et al., 2021). ②. The healing process is initiated when platelets aggregate and release plenty of soluble mediators. Inflammatory responses begin shortly thereafter. When local penetration of neutrophils, lymphocytes, and monocytes increases, blood vessels dilate, capillary permeability increases, and macrophages accumulate and migrate to the wound to promote phagocytosis of the bacteria and damage tissues. The hemostatic and inflammatory phases usually take 3 days to end (Zhang et al., 2020). If this phase is prolonged, too many activated cells are recruited to the injury site, which can harm the wound healing process. ③. The proliferative stage begins within a few days of injury. It is the primary wound healing process and is coordinated by successive but overlapping events such as angiogenesis, collagen deposition, granulation tissue formation, and epithelialization (Pignet et al., 2021). During this stage, fibroblasts synthesize collagen and matrix materials (proteoglycan and fibronectin) to support the formation of new cells and fresh granulation tissue and repair epidermal defects. When fibroblasts are activated and differentiated into myofibroblasts, the excessive myofibroblasts form scars with the occurrence of angiogenesis at this time. Epidermal stem cells migrate linearly to the wound center following epithelialization, and epithelial cells migrate into granulation tissue to close the epidermal deficiency (Yang Z. et al., 2021; Guillamat-Prats, 2021; Monika et al., 2021; Qiang et al., 2021). ④. The remodeling is the last stage. During this stage, collagen type III is replaced by collagen type I to maximize strength in the wound. The increase in tensile strength may last up to a year (Han et al., 2017; Wang P. H. et al., 2018; Lee and Jang, 2018).

Typically, the body is efficient in repairing the skin after injury, yet wound healing is impaired when the normal repair response is out of whack. When a wound fails to heal, tissue recovery is out of kilter, leading to scarring. When the normal repair response goes awry, there are two primary outcomes: ulcerative skin defect (chronic wound) or hyperscarring (HS or keloid) (Eming et al., 2014). Chronic wounds are defined as wounds that cannot be repaired in an orderly and timely manner to achieve anatomic and functional integrity (Atkin, 2019). HS occurs after surgery, trauma, and especially burns. It is usually a hard and red raised scar (Han et al., 2017; Le and Wu, 2021). HS are fibrous scars caused by abnormal skin wound healing processes and are characterized by abnormal proliferation of fibroblasts and excessive deposition of collagen (Wang X. et al., 2018). HS and keloid are the same fibrous hyperplasia skin disease but differ in the intensity and duration of inflammation (Ogawa et al., 2016).

## 3 Therapeutic methods for HS

Currently, the clinical treatment methods for HS include physical therapy, laser therapy, blocking therapy, radiotherapy, surgery, drug therapy, etc. However, physical therapy, laser therapy, glucocorticoid injection, surgical resection, and other treatments are treaty-dependent, with many adverse reactions and even new injuries. Drug therapy has the effect of improving scars. However, the side effects of skin atrophy, capillary dilatation, and local necrosis cannot be ignored when common drugs such as 5-fluorouracil and corticosteroids are used. The current treatment methods for HS are summarized in Table 1.

**TABLE 1:** Current treatment methods for HS.

Therapies	Method	Advantages	Disadvantages
**Pressure therapy**	Continuous pressure on the wound healing site	Non-surgical treatment Li et al. (2018)	Dependence on empirical observation Van den Kerckhove et al. (2005)
		Improvement of scar flexibility Carney et al. (2017)	No standardized applications Kim et al. (2015)
**Laser therapy**	CO_2_ laser	Minimal invasion and low risk Klifto et al. (2020)	pigmentation, hypopigmentation, blister formation and postoperative purpura Klifto et al. (2020)
	Pulsed dye laser	Improvement of the function of scars, including viscoelastic deformity, elastic deformity, and skin roughness Madni et al. (2019)	
**Cryotherapy**	Freezer	Alleviation of clinical scar symptoms Har-Shai et al. (2003)	The high rate of hypopigmentation, skin atrophy, and hyperpigmentation Meymandi et al. (2016)
**Drug therapy**	Triamcinolone acetonide	Improvement in the scar color, thickness, softness, and vascular distribution Song et al. (2019)	Capillary dilatation and pigmentation Waibel et al. (2019)
	5-FU	Mitigation of scar size, color, and texture. Elimination of the pain and itching Goldan et al. (2008)	Pain, burning, ulcers, pigmentation, and skin atrophy Tawfik et al. (2019)
	tacrolimus	Reduce of mucin and improvement of collagen fiber quality and elastic fiber density Menezes et al. (2021)	N.A.

### 3.1 Pressure therapy

Pressure therapy is a non-surgical method to prevent and control HS after the burn (Li et al., 2018). Pressure therapy is a method to suppress scar hyperplasia and promote scar maturation by continuous compression of wound healing parts. Reasonable pressure can reduce the thickness and increase the softness of the scar (DeBruler et al., 2020). PI3K/Akt signaling pathway was found to be an essential regulatory pathway during pressure treatment of HS(Liu et al., 2018). In the study of pressure therapy on the porcine HS model, it was observed that the percentage of elastin continuously increased after pressure treatment during 126 days, while no similar increase was observed in sham scars without pressure treatment. The increased number of elastin is associated with the increased flexibility of scars after stress therapy (Carney et al., 2017). However, the pressure and time in stress therapy still depend on experience (Van den Kerckhove et al., 2005). The lack of standardized applications reduces the effectiveness of stress therapy (Kim et al., 2015). When using a pressure suit for treatment, the design of the pressure suit will affect the contact pressure on the scar (Engrav et al., 2010). The pressure exerted by the pressure clothing on the HS directly affects the therapeutic effect. Therefore, in the research of scar pressure treatment, the precise manufacture of pressure available for specific treatment pressure still deserves further investigation (Leung et al., 2010).

### 3.2 Laser therapy

Laser therapy is a minimally invasive and low-risk treatment that can reduce the neuropathic pain caused by scar and improve the scar function (pigmentation, flexibility, texture, heat sensitivity, and contracture) to improve the overall quality of life of patients (Klifto et al., 2020). CO_2_ laser treatment is one of the comprehensive treatment methods for HS. The pigmentation, blood vessels, flexibility, and scar height were relieved with the decrease of the pain and itching after CO_2_ laser treatment (Zhang et al., 2021). Clinical studies verified the efficacy of the CO_2_ laser treatment. The elasticity and thickness of hypertrophic burn scar were mitigated, and these improvements were stable at least 6 months after the CO_2_ laser treatment (Miletta et al., 2021). Another study of 102 patients with keloid and HS found that 1064 nm Nd:YAG laser in treating HS was significantly better than keloid (Koike et al., 2014). After the treatment with 2940 nm Er:YAG laser, the scars were improved considerably in viscoelastic deformity, elastic deformity, and skin roughness. Such changes were not observed in the untreated scars (Madni et al., 2019). However, laser treatment could also bring side effects such as pigmentation, hypopigmentation, blister formation, and postoperative purpura (Alster, 2003; Chan et al., 2004).

### 3.3 Cryotherapy

Cryotherapy is to destroy scar tissue cells and blood microcirculation at extremely low temperatures to make them necrotic and fall off. Meanwhile, it can lead to scar tissue edema, increased cell space, and decreased scar density (van Leeuwen et al., 2014; Salem et al., 2021). The liquid nitrogen was imported into the freezing probe, and the freezing needle was inserted into the long axis of the HS to freeze the HS. After the HS was completely frozen, the thawed freezing probe was taken out, and the scar volume was reduced by an average of 51.4% after cryosurgery. Cryotherapy could significantly relieve scar symptoms (Har-Shai et al., 2003). However, the incidence of complications during cryotherapy could not be ignored (Meymandi et al., 2016).

### 3.4 Drug therapy

The efficacy of corticosteroid injection in treating HS has been well recognized, yet triamcinolone acetonide has side effects such as capillary dilatation and pigmentation (Waibel et al., 2019). Most HS’s color, thickness, softness, and vascular distribution were mitigated after intralesional injection of triamcinolone acetonide (Song et al., 2019). 5-Fluorouracil (5-FU), a nucleotide analog and a chemotherapeutic drug, can replace uracil with DNA and inhibit DNA synthesis, especially in rapidly proliferating cells. A study reported a patient with obvious HS and keloids in the nasolabial fold, the chin’s front edge, and the mandible’s lateral lower edge. After 2.5 months of treatment with local silica gel tablets and methylprednisolone acetate (40 mg/ml) in acne, the color and size of the lesions were slightly improved without an improvement in the scar fiber structure, the pain, and itching symptoms. After 7 months of 5-FU injection, the scar size, color, and texture were significantly attenuated with complete elimination of the pain and itching (Goldan et al., 2008). However, injection of 5-FU has adverse effects on normal cells with a high proliferative rate, such as digestive epithelial cells, and may also cause leukopenia and thrombocytopenia (Haurani et al., 2009; Yang B. et al., 2021). Other side effects such as pain, burning sensation, ulcer, pigmentation, and skin atrophy frequently occur (Tawfik et al., 2019). Researchers applied tacrolimus to HS to explore whether tacrolimus could promote cell apoptosis and inhibit fibroblast activity. Tacrolimus (0.1%) had a potent inhibitory effect on smooth muscle actin and can reduce the density of collagen fibers scars with the alleviation of scar inflammation even at the concertation of 0.03% tacrolimus. Tacrolimus could effectively inhibit TGF-β Smooth muscle actin, reduce mucin, and enhance collagen fiber quality and elastic fiber density (Menezes et al., 2021).

Many studies have proved that combination therapies have better efficacy than monotherapy. Compared with corticosteroids alone, corticosteroids combined with botulinum toxin A were more effective in treating keloids and HS(Tawfik et al., 2019). Among YAG laser alone, YAG laser combined with intralesional botulinum toxin type A, and YAG laser combined with intralesional steroid injection in the treatment of HS, YAG laser combined with intralesional steroid injection had the highest efficacy and safety in HS patients (Rahman et al., 2021). The HS intense pulsed light combined with lattice CO_2_ laser was also a successful therapeutic combination for HS with significant improvements in the color and texture of scars (Daoud et al., 2019). In summary, compared with monotherapy, the combination therapy makes the treatment more effective with less trauma, fewer side effects, a low recurrence rate, and a short course of treatment.

## 4 Effects of TCM on HS

In recent years, more and more botanical compounds exacted from TCM are discovered and verified as drug candidates for treating HS. The botanical sources of TCM included Alpinia officinarum Hance, *Centella asiatica* (L.) Urb., Rheum palmatum L., Panax ginseng C.A.Mey., Scutellaria baicalensis Georgi*,* Ginkgo biloba L.*,* Conioselinum anthriscoides ‘Chuanxiong’, Salvia miltiorrhiza Bunge, Taxus wallichiana Zucc., Stephania tetrandra S. Moore, and Kaempferia galanga L.

Galangin (3,5,7-trihydroxyflavone) is a botanical compound extracted from the root of Alpinia officinarum Hance. *In-vivo* studies have shown that it had a potent anti-inflammatory effect using rabbit ear HS model. Galangin inhibited HS formation through the TGF-β/Smad signaling pathway (Ru et al., 2021) and ALK5/Smad2/3 signaling pathway (Zhang Y. et al., 2016). These results suggest that *galangal* is a potential drug candidate for treating HS.

Asiaticoside is extracted from *Centella asiatica* (L.) Urban. It has been used to treat skin, venous, and microvascular diseases for many years. Asiaticoside increased the mobility of skin cells and enhanced the adhesion of initial skin cells in the wound suture seeding model. The diffusion and migration of skin cells were the main determinants of wound healing, suggesting that asiaticoside could be used as a potential promoter of wound healing (Lee et al., 2012). Oral administration of asiaticoside at 24 mg/kg/d to rabbits remarkably attenuated wound healing, and reduced scar thickness, thereby inhibiting HS(Huang et al., 2021). Asiaticoside inhibited the increase of Smad7 expression by interrupting TGF-β signal transmission through negative feedback and significantly alleviated HS in rabbit ears (Ju-Lin et al., 2009).

As one of the main components of Rheum palmatum L., emodin is widely used in treating inflammatory and non-inflammatory diseases (such as cancer). The potential therapeutic effects of emodin in HS have been elucidated (Li W. et al., 2021; Jiang et al., 2021). The researchers used the mouse HS model induced by mechanical stress to study the effect of emodin on HS. The results indicated that emodin (40 mg/ml) had a therapeutic effect on the formation of HS, which was potentially related to the inhibition of the PI3K/Akt signal pathway (Liu, 2015). Further *in vitro* experiments verified that emodin modulated the polarization of macrophages M1 and M2 to reduce the formation and fibrosis of HS through the Notch and TGF-β pathways (Xia et al., 2021).

Ginsenoside Rb1 is extracted from the Panax ginseng C.A.Mey. It promoted angiogenesis to improve burn wound healing in the process of skin wound repair in mice (Kimura et al., 2006). Ginsenoside Rb1 also inhibited MMP2, TIMP1, α-SMA, and TGF-β1 to alleviate HS in rabbits (Tark et al., 2015). Besides, ginsenoside Rg3, with two optical isomers of 20 (R)—Rg3 and 20 (S)-Rg3, showed the highest HS inhibitory efficacy among ginsenosides. Studies revealed that 20 (R)-Rg3 could inhibit HS in *in vitro* model (HS specimens from patients) through the TGF-β/Smad and ERK1/2 signal pathways (Tang et al., 2018), inferring that 20 (S)-Rg3 could be potentially used as an early intervention to reduce the formation of HS.

Baicalin is a flavonoid compound extracted from the roots of Scutellaria baicalensis Georgi (Li et al., 2019). After treatment with baicalin, the formation of HS in mice model induced by mechanical load was remarkably reduced. The mechanism of the inhibitory effect of baicalin on HS was related to the TGF-β/Smad2/3 signaling pathways (Zhang Y. F. et al., 2016). Also, baicalin regulates the miR-9/IGF-1 axis to inhibit cell proliferation and collagen production through NF-κB and Wnt/β-catenin signaling pathways (Yang L. et al., 2019).

Flavonoid quercetin is a heterocyclic polyphenol. Quercetin exists in Ginkgo biloba L. and various fruits and vegetables. It has antiviral, anti-inflammatory, and antibacterial activities (Song J. Y. et al., 2018). *In-vitro* studies testified that quercetin suppressed scar formation by inhibiting proliferation and contraction of excessive scar-derived fibroblasts (Phan et al., 2003).

Moreover, the essential oil from Conioselinum anthriscoides ‘Chuanxiong' (Wu et al., 2011), cryptotanshinone from Salvia miltiorrhiza Bunge (Li Y. et al., 2016), paclitaxel from Taxus wallichiana Zucc.(Huang L. P. et al., 2015), tetrandrine from Stephania tetrandra S. Moore (Ning et al., 2016), and kaempferol from Kaempferia galanga L.(Li H. et al., 2016) could mitigate the formation of HS (Table 2).

**TABLE 2:** A summary of the commonly used TCM in the treatment of HS.

Sources	Component/Extracts	*In vivo* model	*In vitro* model	Dose	Duration	Minimal active concentration	Controls	Signaling pathways	Effects	References
Alpinia officinarum Hance	Galangin	Rabbit ear HS model	N.A.	2, 1 or 0.5 mg/ml/3days	12 days	0.5 mg/ml	Negative (Saline)	TGF-β/Smad signaling pathway	Inhibition of HS formation	Ru et al. (2021)
		BALB/c mice HS model	HS fibroblasts from patients	*In vivo* (10 μM/day)	*In vivo* (17 days)	*In vivo* (10 μM)	Negative (DMSO)	ALK5/Smad2/3 signaling pathway	Attenuation of HS formation	Zhang et al. (2016a)
				*In vitro* (0.1–25 μM)	*In vitro* (5 days)	*In vitro* (N.A.)				
*Centella asiatica* (L.) Urb	Asiaticoside	N.A.	A wound closure seeding model	N.A.	5 days	62.5 μM	Negative (DMSO)	N.A.	Increase of skin cell mobility and initial skin cell adhesion	Lee et al. (2012)
		Rabbit ear HS model	N.A.	12 or 24 mg/kg/day	60 days	N.A.	Negative (water)	N.A.	Inhibition of HS formation	Huang et al. (2021)
		Rabbit ear HS model	N.A.	0.5% or 1%	60 days	N.A.	Negative (Saline)	TGF-β/Smad7 signaling pathway	Alleviation of HS	Ju-Lin et al. (2009)
Rheum palmatum L	Emodin	C57BL/6 mice HS model		20, 40, 80 or 120 mg/ml	24 days	40 mg/ml	Negative (Dulbecco’s modified Eagle medium)	PI3K/Akt signaling pathway	Alleviation of HS formation	Liu, (2015)
		A rat tail HS model and dorsal subcutaneous polyvinyl alcohol sponge-induced wounds	N.A.	10 mg/kg/day	42 days	N.A.	Negative (Dulbecco’s modified Eagle medium)	Notch and TGF-β signaling pathway	Alleviation of HS formation	Xia et al. (2021)
Panax ginseng C.A.Mey	Ginsenoside Rb1	A burn wound of mice	HaCaT cells	*In vivo* (100, 1, 10 pg/g)	*In vivo* (19 days)	*In vivo* (100 pg/g)	*In vivo* (Negative: Vaseline)	N.A.	Promotion of wound healing	Kimura et al. (2006)
				*In vitro* (100 fg/ml to 1 ng/ml)	*In vitro* (N.A.)	*In vitro* (N.A.)	*In vitro* (Negative: Dulbecco’s modified Eagle medium)			
		Rabbit ear HS model	N.A.	0.01, 0.04, 0.08 mg/day	7 days	0.1 mg/ml	Negative (Saline)	N.A.	Inhibition of HS	Tark et al. (2015)
	Ginsenoside 20 (R)-Rg3	N.A.	HS specimens from patients	50 or 100 μg/ml	5 days	50 μg/ml	Negative (Dulbecco’s modified Eagle medium)	TGF-β/SMAD and ERK1/2 signaling pathway	Inhibition of HS	Tang et al. (2018)
Scutellaria baicalensis Georgi	Baicalin	BALB/c mice	Human HS-derived fibroblasts	*In vivo* (0.001 μM)	*In vivo* (24 days)	*In vivo* (10 μmol/L )	Negative (DMSO)	TGF-β/Smad2/3 signaling pathway	Alleviation of HS formation	Zhang et al. (2016b)
				*In vitro* (1, 5, or 10 μM)	*In vitro* (N.A.)	*In vitro* (N.A.)				
		N.A.	NIH/3T3 cells	150 μM	N.A.	150 μM	Negative (Dulbecco’s modified Eagle medium)	NF-κB and Wnt/β-catenin Signaling pathway	Alleviation of HS formation	Yang et al. (2019a)
Ginkgo biloba L	Quercetin	N.A.	Fibroblasts from patients	5–20 μg/ml	N.A.	5 μg/ml	Negative (Dulbecco’s modified Eagle medium)	TGF-β/Smad signaling pathway	Inhibition of HS formation	Phan et al. (2003)
Conioselinum anthriscoides ‘Chuanxiong'	Ligusticum chuanxiong	Rabbit ear HS model	N.A.	5%, 10%, and 20%	22 days	5%	Negative (vaseline and liquid paraffin) positive (contracture)	N.A.	Inhibition of HS formation	Wu et al. (2011)
Salvia miltiorrhiza Bunge	Cryptotanshinone	Wound of Balb/c mice	HS tissues from patients	*In vivo* (25 ng/g)	*In vivo* (14 days)	*In vivo* (25 ng/g)	*In vivo* (Negative: Saline)	N.A.	Promotion of wound healing	Li et al. (2016b)
				*In vitro* (12.5 or 25 μM)	*In vitro* (24 or 48 h)	*In vitro* (12.5μM)	*In vitro* (Negative: DMSO)			
Taxus wallichiana Zucc	Paclitaxel	Rabbit ear HS model	N.A.	0.096mg–30 mg	10 days	48 mg/L	Negative (Saline)	N.A.	Inhibition of HS formation	Huang et al. (2015a)
Stephania tetrandra S. Moore	Tetrandrine	N.A.	Human HS fibroblasts	5 mg/ml	48 h	5 mg/ml	Negative (Dulbecco’s modified Eagle medium)	Hsa-miR-125 b and Hsa-miR-27 b	Inhibition of HS formation	Ning et al. (2016)
Kaempferol galanga L	Kaempferol	A mouse HS model	Fibroblasts from patients	*In vivo* (10 μM)	*In vivo* (17 days)	*In vivo* (10 μM)	Negative (DMSO)	TGF-β1/Smads Signaling pathway	Inhibition of HS formation	Li et al. (2016a)
				*In vitro* (1, 5, 10 or 25 μM)	*In vitro* (N.A.)	*In vitro* (1 μM)				

## 5 The HS inhibitory mechanism of TCM

In recent years, *in vivo* and *in vitro* studies have found that the mechanism of TCM inhibiting HS can be divided into reducing the inflammatory response, inhibiting fibroblast proliferation, inducing fibroblast apoptosis and autophagy, promoting the degradation of extracellular matrix, reducing angiogenesis, and inhibiting cutaneous nerve system.

### 5.1 Reduction of inflammation

The potential mechanism of wound healing is complex. So far, inflammation is one of the admitted decisive factors. Inflammation occurs after skin injury due to tissue damage and microbial invasion. HS is a pathological scar caused by abnormal wound healing and is characterized by persistent local inflammation and excessive collagen deposition.

**FIGURE 2:**
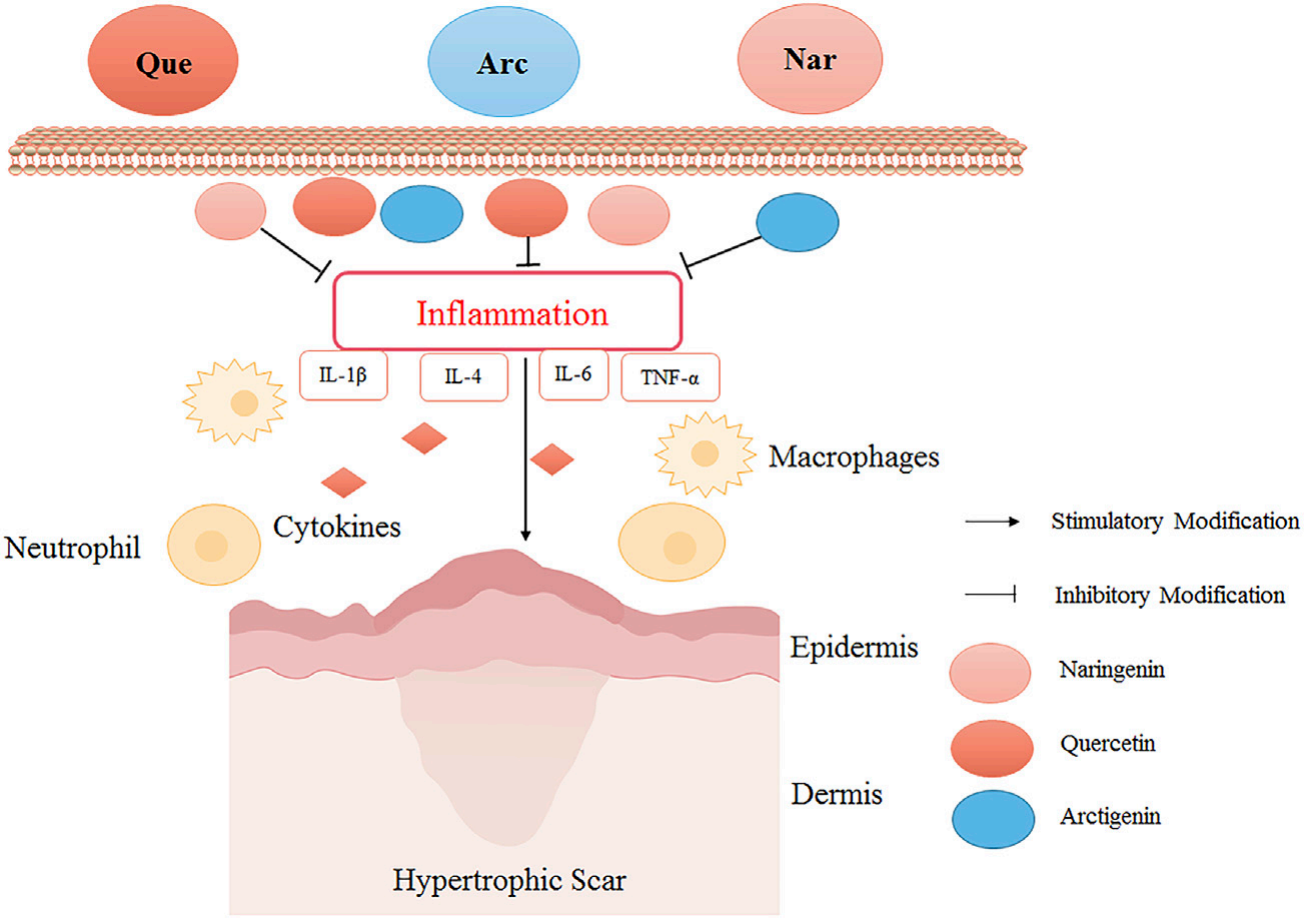
The HS inhibitory mechanism of TCM (quercetin, arctigenin, and naringenin) by reducing inflammation. Quercetin (Que), arctigenin (Arc), and naringenin (Nar) could inhibit the infiltration of inflammatory cells and the production of inflammatory cytokines to alleviate HS. Quercetin reduced the number of macrophages in the skin wound healing process, relieved inflammation, and reduced HS formation. Arctigenin alleviated HS by reducing inflammatory factors IL-1β, IL-4, IL-6, and TNF-α. Naringin down-regulated inflammatory cytokines to exert its anti-inflammatory effect to alleviate HS.

Quercetin could reduce the number of macrophages and myofibroblasts during wound healing in rabbits, thereby reducing HS formation (Song J. Y. et al., 2018). Arctigenin was used in a bleomycin-induced mouse model of skin fibrosis. The results showed that the expression levels of IL-1β, IL-4, IL-6, TNF-α, and monocyte chemoattractant protein decreased significantly after arctigenin treatment, inferring that arctigenin attenuated HS potentially by reducing inflammatory response (Jiang et al., 2020). In the mouse model of HS induced by mechanical stretching (Shan et al., 2017), naringenin effectively suppressed the infiltration of inflammatory cells and the production of inflammatory cytokines (TNF-α, IL-1β, and IL-6), thereby exerting its anti-inflammatory effect to alleviate HS (Figure 2).

### 5.2 Inhibition of fibroblast proliferation and induction of fibroblast apoptosis and autophagy

*In-vitro* studies primarily focus on fibroblasts in the scar due to they have more active proliferation and invasion ability than fibroblasts in the steady-state environment. The activity of myofibroblasts in the scar is much higher than that in the normal state.

**FIGURE 3:**
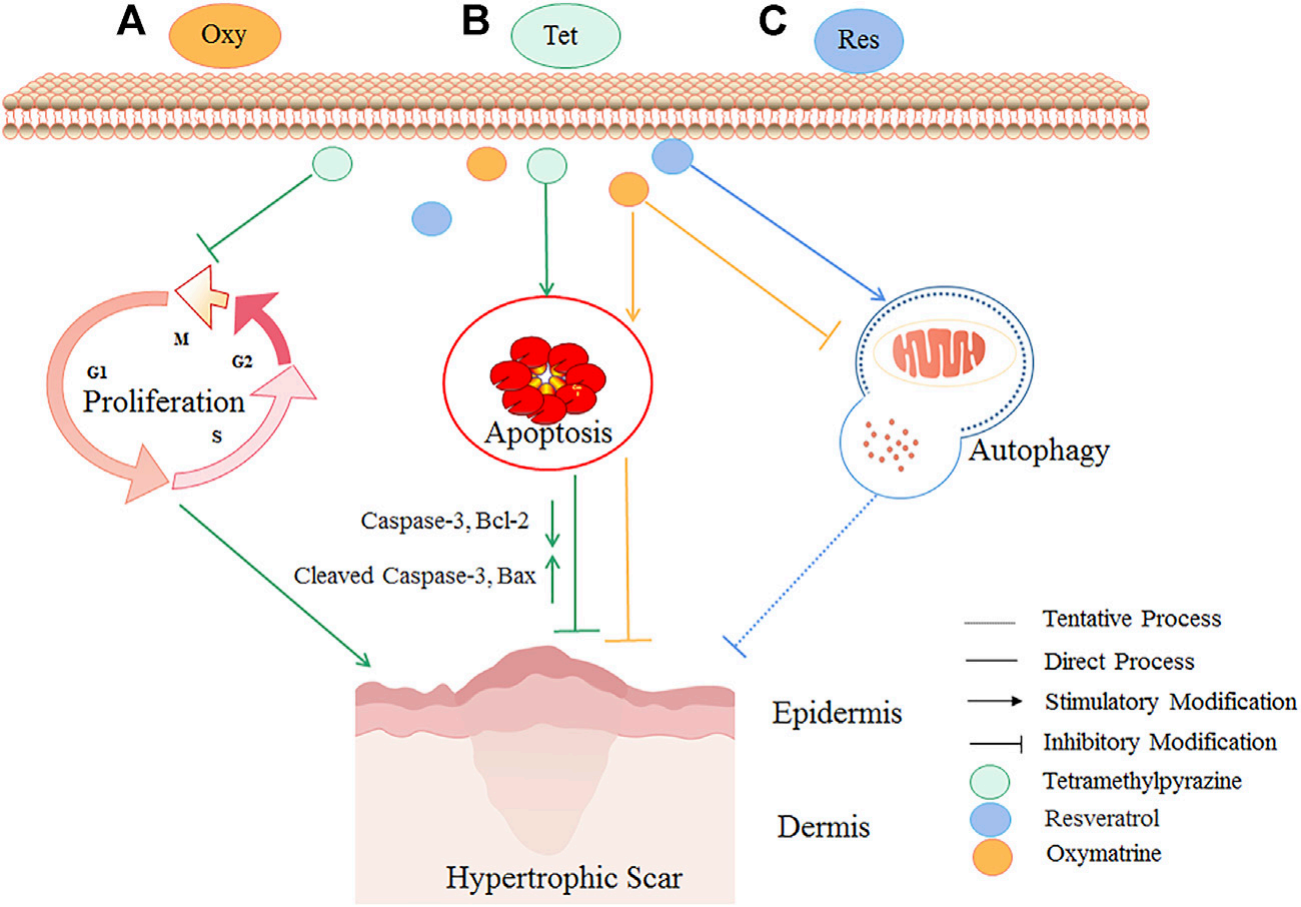
The HS inhibitory mechanism of TCM (oxymatrine, tetramethylpyrazine, and resveratrol) by inhibiting fibroblast proliferation and inducing fibroblasts’ apoptosis and autophagy. Autophagy is a conserved catabolic pathway that maintains cell metabolism and homeostasis. However, excessively activated autophagy can lead to cell death. Oxymatrine **(A)** could reduce scar area and epidermal-dermal thickness potentially by inhibiting autophagy and inducing apoptosis. Tetramethylpyrazine **(B)** could attenuate HS by inhibiting the proliferation of scar fibroblasts, activating the expression of proapoptotic proteins Bax and Cleaved Caspase-3, and reducing Caspase-3 and Bcl-2. Resveratrol **(C)** could potentially treat HS by activating autophagy. The yellow lines are the cellular processes modulated by oxymatrine, the green lines are the cellular processes modulated by tetramethylpyrazine, and the blue lines are the cellular processes modulated by resveratrol.

*In-vitro* studies have found that oxymatrine reduced the activity and collagen metabolism of human scar fibroblasts, increased cell apoptosis, and reduced the scar area and thickness (Deng et al., 2021). Tetramethylpyrazine, the primary chemical component of Conioselinum anthriscoides “Chuanxiong” down-regulated fibrosis-related molecules (type I collagen, type III collagen, and α-SMA) to inhibit the proliferation of scar fibroblasts and activated the expression of Bax and Cleaved Caspase-3, which finally promoted the repair of HS(Wu et al., 2020). Besides, resveratrol inhibited HS formation by activating autophagy in HS fibroblasts *via* the miR-4654/Rheb axis (Pang et al., 2020). As shown in Figure 3, some botanical drugs can inhibit fibroblast proliferation and induce fibroblasts’ apoptosis and autophagy to treat HS.

### 5.3 Degradation of extracellular matrix

In the remodeling stage of wound healing, the imbalance of matrix-degradation enzymes will cause excessive collagen synthesis and abnormal collagen conversion, resulting in exceeding wound healing, that is, scar formation. Therefore, promoting the extracellular matrix’s degradation can reduce the scar’s occurrence.

**FIGURE 4:**
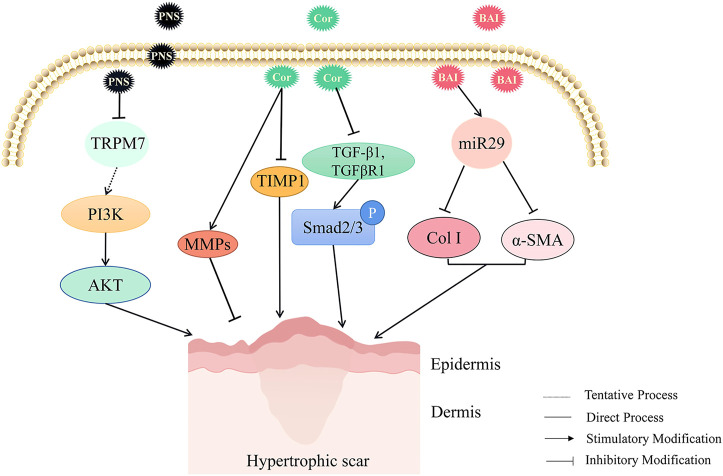
The HS inhibitory mechanism of TCM (Panax notoginseng saponins, corilagin, and baicalein) by promoting extracellular matrix degradation. Panax notoginseng saponins (PNS), corilagin (Cor), and baicalein (BAI) could promote the degradation of the extracellular matrix to reduce HS. PNS inhibited HS formation potentially by inhibiting TRPM7 and regulating PI3K/AKT signaling pathway to inhibit extracellular matrix deposition. Cor inhibited TGF-β1, reduced the expression of p-Smad2/3, affected the levels of MMPs and TIMP1 proteins, inhibited the deposition of extracellular matrix, and thus attenuated HS. Up-regulation of miR-29 by BAI could inhibit TGFβ1-induced accumulation of total soluble collagen, collagen I, and α-SMA to alleviate HS.

Panax notoginseng saponins (PNS) inhibited the extracellular matrix synthesis, stimulated cell apoptosis, and regulated the PI3K/AKT signaling pathway by changing the expression of TRPM7, thus hindering scar formation (Zhi et al., 2021). Corilagin (Cor) is a kind of ellagic tannin that exists in *Phyllanthus* Emblica L. and *Geranium* robertianum L. It has been found that in the rabbit ear scar model, corilagin could affect the protein levels of MMPs (matrix metalloproteinases) and TIMP1 (tissue inhibitor of metalloproteinases), inhibit the protein expression of TGF-β1, and reduce the level of p-Smad2/3, thereby inhibiting the deposition of extracellular matrix and various functions of fibroblasts. It is a potential drug for the treatment of HS(Li et al., 2021b). Baicalein (BAI) also effectively inhibited cell proliferation by inhibiting TGF-β1-induced accumulation of total soluble collagen, collagen I, and α-SMA through up-regulating miR29 (Yang X. et al., 2019) (As shown in Figure 4).

### 5.4 Inhibition of angiogenesis

Angiogenesis plays a vital role in wound healing, which involves the proliferation, migration, and formation of endothelial cells. Studies have shown that the number of microvessels in HS increases (Zheng et al., 2014; Molina et al., 2022).

Usnic acid has a variety of biological activities, such as antiviral, anti-microbial, anti-inflammatory, and anti-proliferation. Usnic acid could significantly inhibit the formation of HS and considerably reduce the thickness and color of the scar in the rabbit ear model. It has been proved that usnic acid remarkably inhibited scar angiogenesis using immunohistochemical analysis of CD31 expression. It also inhibited the proliferation of human umbilical vein endothelial cells and scar fibroblasts. These results proved that the therapeutic effect of usnic acid on HS formation in rabbit ears was due to its inhibition of scar angiogenesis (Song Y. et al., 2018). Another study revealed that a biflavonoid compound amentoflavone extracted from Selaginella tamariscina (P.Beauv.) spring also significantly inhibited the viability, migration, and angiogenesis of endothelial cells related to angiogenesis to treat HS(Zhang et al., 2014).

### 5.5 Inhibition of cutaneous nerve system

Inflammation and the cutaneous nerve system play essential roles in wound healing and HS formation (Yang et al., 2014; Li et al., 2015; Zhang et al., 2022). The evidence that cutaneous neurogenic inflammation could stimulate fibroblasts during scar formation has been confirmed (Zhang et al., 2022). Neurogenic inflammation is an inflammatory process due to acute injury with the release of neuropeptides, especially neuropeptide substance *p* (SP), from sensory nerves (Black, 2002). It has been proved that the concentration of SP was significantly increased in HS(Scott et al., 2007). Thus, the SP with its related signaling pathways may be a novel therapeutic target for HS.

SP and IL-33 released by nerves could significantly increase the secretion and gene expression of pro-inflammatory cytokine IL-1β by activating their corresponding receptors NK-1 and ST2 on human mast cells. Natural flavonoid methoxy luteolin could effectively reverse this trend and attenuate the inflammatory response, providing new therapeutic targets for treating inflammatory diseases (Taracanova et al., 2018). Puerarin, a single flavonoid glycoside, prevented paclitaxel-induced peripheral neuropathic pain in rats by inhibiting the upregulation of SP, TRPV1, and calcitonin gene-related peptide (Kong et al., 2015; Wu et al., 2019), indicating the inhibitory effects and potential of flavonoids on SP. Besides, many researches have proven the potential of flavonoids in the treatment of HS, including kaempferol (Li H. et al., 2016), quercetin (Song J. Y. et al., 2018), and dihydromyricetin (Ye et al., 2019). Can flavonoids alleviate HS by inhibiting cutaneous neurogenic inflammation *via* SP as the therapeutic target? Experimental evidence is needed. However, this provides a new idea for the mechanism study of flavonoids in treating HS.

## 6 New drug delivery system of TCM for HS

Many botanical drugs extracted from TCM have an anti-HS effect. However, the poor solubility and absorption of botanical drugs and the skin’s natural barrier hindered the penetration of chemical components and reduced their therapeutic effect (Ning et al., 2021). Long-term sustained-release and targeted preparations of TCM with appropriate carriers provide a new idea for the application of TCM in scar treatment. More and more new TCM delivery systems are developed, including hydrogel, microneedle, nano, liposome, etc.

### 6.1 Hydrogel

Hydrogels with different gelation mechanisms and compositions are one of the most widely used biomaterials (Sultan et al., 2019). Their advantages include good biocompatibility, ease to use, low toxicity, and excellent stability (Matiasek et al., 2018).

Researchers have prepared a new hydrogel composed of poly-γ-PGA, chit oligosaccharide, and papain. This hydrogel had a predominant porous three-dimensional network structure, good water absorption performance, and mechanical properties. This hydrogel promoted cell adhesion and inhibited the excessive proliferation of fibroblasts, which had the potential for *in vivo* application. It has been testified that this hydrogel could effectively inhibit excessive collagen deposition and HS formation during wound healing (Xue et al., 2021). The drug-loaded hydrogel using anti-HS botanical drugs may be a feasible preparation to increase the efficacy of the treatment of HS.

### 6.2 Microneedle

Microneedles can penetrate the cuticle barrier of the skin and quickly establish a large number of micro-dermal channels to make the drug penetrate and absorb accurately, which not only avoids the first pass effect of the liver but also has the advantages of no damage and no pain (McCrudden et al., 2015).

**FIGURE 5:**
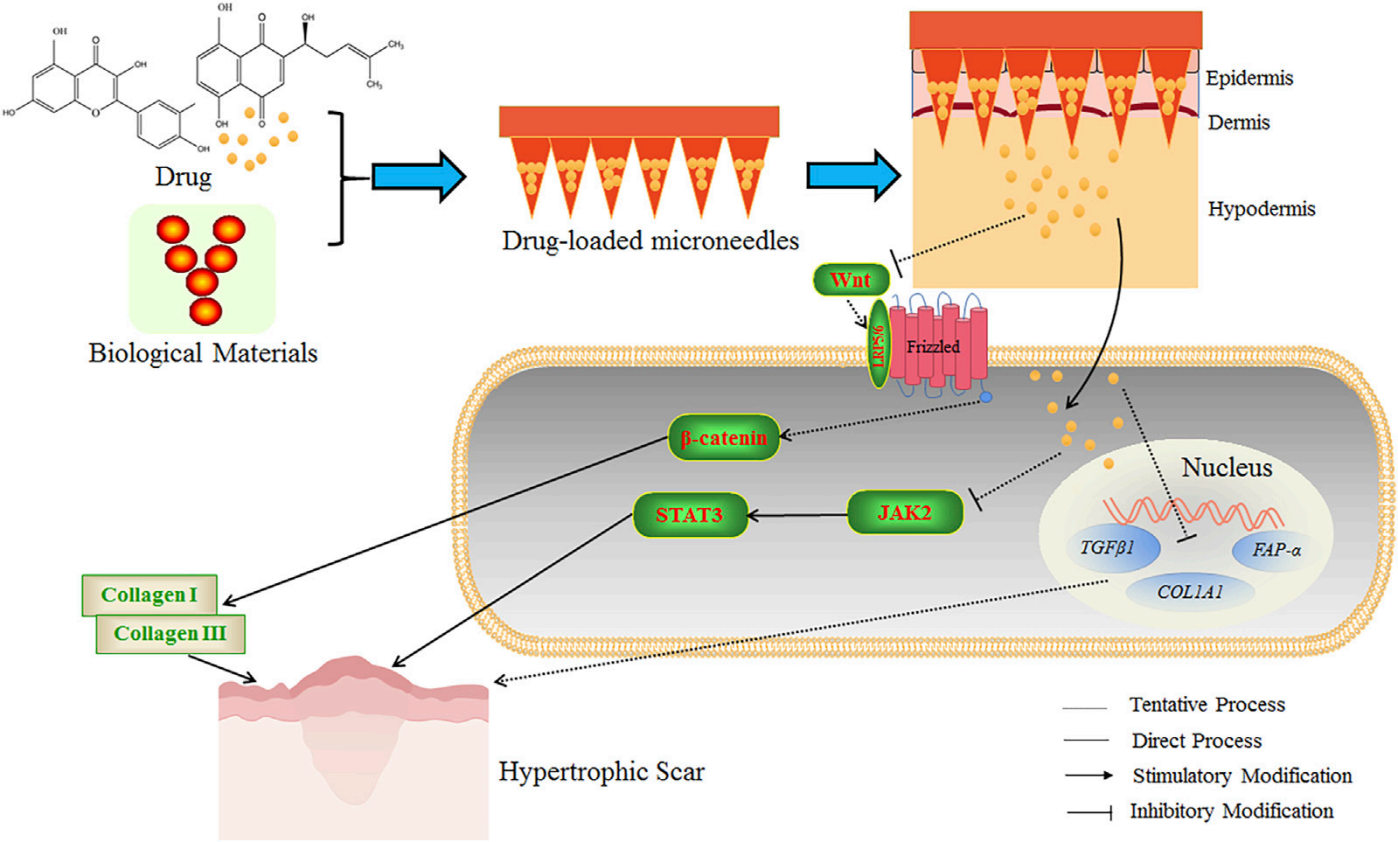
The schematic diagram of drug-loaded microneedles for the treatment of HS. Microneedles were prepared using biological materials and botanical drugs, which could establish many micro-dermal channels to accurately promote the penetration of drugs into the dermis and hypodermis. The Wnt ligand is a secreted glycoprotein that binds to Frizzled receptors, leading to the formation of a cell surface complex (Wnt-bound Frizzled combined with coreceptor LRP5/6), and β-catenin is subsequently activated and stabilized. The activated β-catenin can stimulate the transcription of Wnt target genes and the expression of fibrosis-related genes and promote the formation of collagen Ⅰ and Ⅲ to increase the HS formation. JAK2/STAT3 signaling pathway is essential in inhibiting apoptosis and tissue fibrosis. STAT3 is highly expressed in HS tissues. The application of Shikonin soluble hyaluronic acid microneedle could enhance the therapeutic effect of HS by down-regulating fibrosis-related genes to reduce the viability and proliferation of fibroblasts. The quercetin-loaded microneedle complex could improve the therapeutic effect on HS potentially by inhibiting the Wnt/β-catenin signaling pathway, reducing STAT3 expression, and inhibiting the JAK2/STAT3 signaling pathway. The above microneedles could improve the anti-HS efficacy of the botanical drugs.

The schematic diagram of the microneedle is shown in Figure 5. The researchers mixed shikonin with hyaluronic acid to produce microneedles with enough mechanical strength to penetrate the skin and controllable dosage during preparation. The shikonin-soluble hyaluronic acid microneedle remarkably attenuated the viability and proliferation of fibroblasts and down-regulated fibrosis-related genes (*TGF-β1, FAP-α, and COL1A1*) to enhance the local therapeutic effect, which was conducive to the treatment of HS(Ning et al., 2021). In addition, other researchers have developed an active targeting drug delivery system for the local treatment of HS. The metal-organic framework of diphenyl carbide crosslinked cyclodextrin containing 26% quercetin was coated on the fibroblast membrane of HS. Then, the Bletilla striata polysaccharide was dispersed to prepare soluble microneedles. This microneedle showed remarkable anti-HS efficacy in rabbits (Wu et al., 2021).

### 6.3 Nano

**FIGURE 6:**
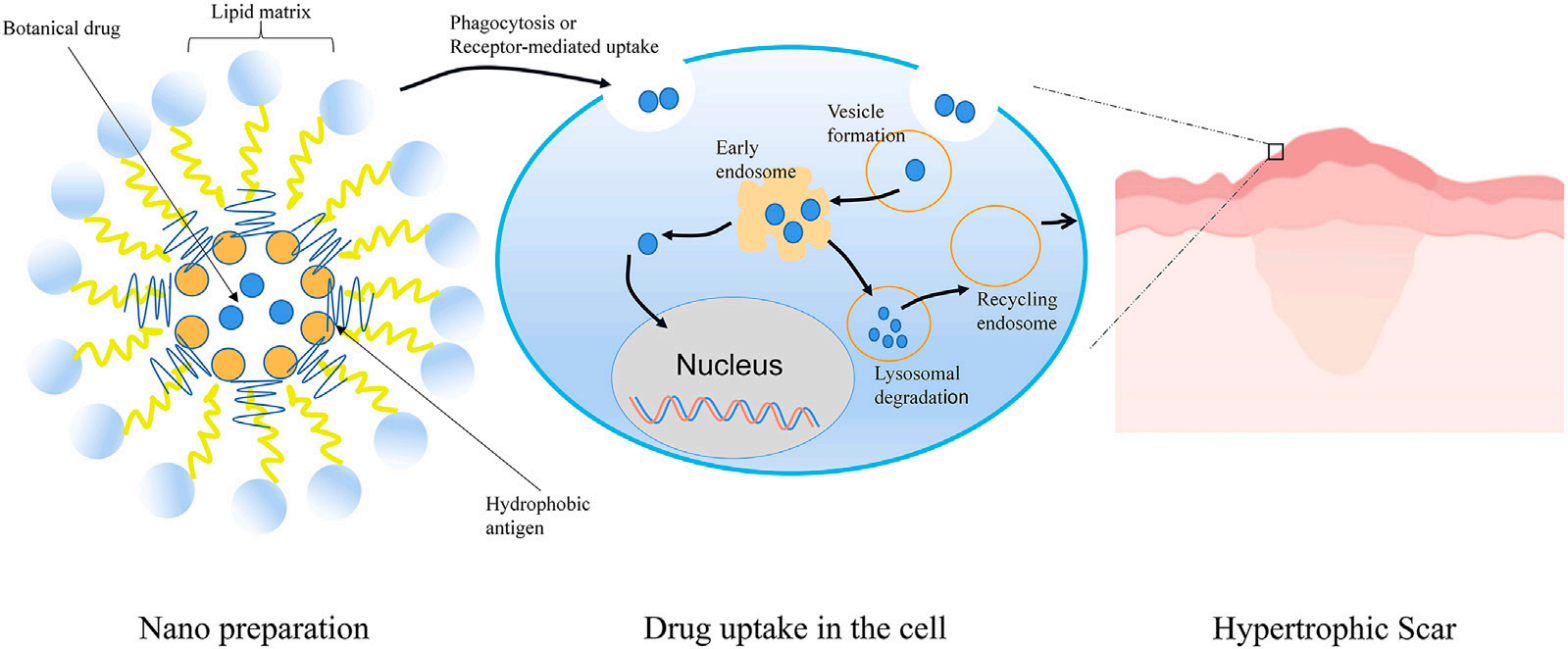
Schematic representation of Nano TCM delivery system to treat HS. Botanical drug-loaded nanoparticles were prepared by the self-assembling approach. The nanoparticles were absorbed into dermal cells by phagocytosis or receptor-mediated uptake. Subsequently, the free drug was released into the cell after the process of endosome and lysosome. Nano TCM delivery system could effectively improve the solubility and permeability of active ingredients in TCM. Spiral spray paeonol nanogel showed good transdermal performance and dermal retention. Asiaticoside nanoparticles, nanoemulsion, and nanoemulsion gel effectively overcame the skin barrier and had high drug permeability to enhance the therapeutic effect of HS.

Nano TCM delivery system can effectively improve the solubility and stability of effective components of TCM, improve bioavailability, and reduce the adverse reactions caused by drugs entering the systemic circulation (Huang Y. et al., 2015) (Figure 6).

Preparation of paeonol nanogel by spiral spray had good transdermal performance and dermal retention without stimulating the skin, providing a potential treatment strategy for HS(Guo et al., 2019). Asiaticoside is a triterpenoid pentacyclic saponin that has a pronounced therapeutic effect on human HS. However, its high molecular weight, low water solubility, and poor lipophilicity are not conducive to diffusion through the stratum corneum. The researchers designed asiaticoside nanoemulsion and nanoemulsion gel and found that these nano delivery systems showed high drug permeability through the transdermal pathway without toxicity (Li et al., 2020).

### 6.4 Other TCM delivery systems

It has been reported that TCM-loaded liposomes could promote drug permeability into heterogeneous scar *epidermis* and enhance dermal drug retention. Cell-penetrating peptide-modified salvianolic acid B liposomes successfully inhibited the proliferation, migration, and invasion of fibroblasts in a concentration- and time-dependent manner, providing a promising therapeutic strategy for transdermal drug delivery (Shi et al., 2020). Besides, microemulsion, a thermodynamic and kinetic stable system, improved the solubility of drugs and had strong transdermal permeable properties. Furthermore, it was proved that microemulsion and oxymatrine-phospholipid complex could enormously enhance the inhibitory effect of oxymatrine on scar fibroblasts (Cao et al., 2011). So far, there are more and more new TCM delivery systems are developed on the way.

## 7 Conclusion

HS is a fibrous tissue proliferative skin disease caused by abnormal proliferation of fibroblasts and excessive collagen deposition. The mechanisms of botanical compounds/extracts from TCM in the treatment of HS, including reducing inflammation, inhibiting fibroblast proliferation, regulating fibroblast activation and migration, inducing fibroblast apoptosis and autophagy, promoting extracellular matrix (ECM) degradation, reducing angiogenesis, and inhibiting cutaneous nerve system, are reviewed in the paper, and the new drug delivery systems of anti-HS botanical compounds are also reviewed, aiming to provide ideas for further research of TCM in the treatment of HS and finally to promote more efficient anti-HS botanical candidates for clinical application. With the updated knowledge on the mechanism of HS and the fast progress of TCM research, the botanical drugs extracted from TCM have made outstanding achievements in preventing and treating HS. However, the limitations should not be ignored. The phytopharmacology research of HS primarily focused on limited pathways without deep mechanism elucidation and new signaling pathways and targets. Interdisciplinary and larger datasets-driven research has been frequently used in pharmacology research, and more and more state-of-the-art sophisticated techniques are arising. The phytopharmacology research level is still far from pharmacology research level. Moreover, the metabolism and stability study and the transdermal kinetic study of botanical compounds in the treatment of HS are rare. Still, many multiherbal preparations (Fufang) in the treatment of HS have been clinically used in China, yet the related studies are limited. Most phytopharmacology studies of HS used a single botanical compound in animal models or cell models, which could not stand for TCM and was hard to extrapolate and lacked clinical relevance. Also, due to the complexity of the effective components in TCM, it still lacks a sufficient theoretical basis to design a reasonable dosage with TCM in clinical application. Thus, the updated analysis technology (such as High-resolution Mass Spectrometry and Ion Mobility Mass Spectrometry) combined with the bioinformatics method, as a priority, should be used to reveal the chemical components in botanical extracts, which will facilitate the pharmacology study and discovery of new candidates from TCM in the treatment of HS. The deep and exact phytopharmacological mechanism study and the new drug delivery system study of the botanical drugs in the treatment of HS based on interdisciplinary techniques should be focused on in the future. The combination of pharmacokinetics and pharmacodynamics, even the deep learning (such as BP/Elman Neural Network), should be also necessary for the phytopharmacology research of HS.
